# The Outcome of Neutrophil-T Cell Contact Differs Depending on Activation Status of Both Cell Types

**DOI:** 10.3389/fimmu.2021.633486

**Published:** 2021-03-30

**Authors:** Danielle Minns, Katie J. Smith, Gareth Hardisty, Adriano G. Rossi, Emily Gwyer Findlay

**Affiliations:** Centre for Inflammation Research, College of Medicine and Veterinary Medicine, University of Edinburgh, Edinburgh, United Kingdom

**Keywords:** neutrophils, T cells, inflammation, culture models, PD1

## Abstract

Neutrophils and T cells exist in close proximity in lymph nodes and inflamed tissues during health and disease. They are able to form stable interactions, with profound effects on the phenotype and function of the T cells. However, the outcome of these effects are frequently contradictory; in some systems neutrophils suppress T cell proliferation, in others they are activatory or present antigen directly. Published protocols modelling these interactions *in vitro* do not reflect the full range of interactions found *in vivo*; they do not examine how activated and naïve T cells differentially respond to neutrophils, or whether de-granulating or resting neutrophils induce different outcomes. Here, we established a culture protocol to ask these questions with human T cells and autologous neutrophils. We find that resting neutrophils suppress T cell proliferation, activation and cytokine production but that de-granulating neutrophils do not, and neutrophil-released intracellular contents enhance proliferation. Strikingly, we also demonstrate that T cells early in the activation process are susceptible to suppression by neutrophils, while later-stage T cells are not, and naïve T cells do not respond at all. Our protocol therefore allows nuanced analysis of the outcome of interaction of these cells and may explain the contradictory results observed previously.

## Introduction

The ability of neutrophils to influence adaptive immune responses is now well-established. Far from being unsophisticated, short-lived cells only involved in killing during acute infection, we now know that neutrophils can survive for up to 5 or 6 days (1), and can orchestrate T cell responses to infection and autoimmunity. For example, neutrophils regulate T cell responses in asthma (2, 3), assist maturation of antigen presenting cells in models of Multiple Sclerosis (4) and alter subset differentiation and cytokine production of tumour-infiltrating T cells (5–8).

Stable interactions between the two cells have been observed (9), with formation of an ‘immune synapse’ which can persist for minutes (9). These interactions have profound effects on the phenotype and function of the T cells. However, the data is confusing; in some studies neutrophils can activate T cells, present antigen directly and enhance proliferation (10–14), while in others neutrophils suppress proliferation and induce apoptosis (9, 15–17). The reasons behind these differential outcomes are currently unclear. It is possible that alterations in the inflammatory milieu in each case, subtly different neutrophil populations, or the presence of different T cell subsets, leads to divergent outcomes; however, this has scarcely been examined.

An extra layer of complexity is provided by the fact that not only intact neutrophils, but also their released mediators, can affect T cell behavior. For example, exocytosis of certain anti-microbial and/or cytotoxic molecules such as myeloperoxidase and arginase-1 have been shown to suppress T cell proliferation (18–20), and we have recently shown that neutrophil-derived cathelicidin can skew T cell differentiation towards a Th17 phenotype and promote survival (21). Furthermore, release of neutrophil extracellular traps (NETs) can directly prime human CD4^+^ T cells by reducing their activation threshold so that they can respond to suboptimal stimulation (22). NETs have been shown to induce Th17 responses in psoriasis (23) and promote type 2 immunity in the lung following rhinovirus infection (3). In HIV/SIV-infected individuals, the overproduction of NETs has also been suggested to induce apoptosis in both CD4^+^ and CD8^+^ T cells (24). These data, together, imply that different modes of neutrophil death may lead to different outcomes in local T cells; again, this and the mechanisms underlying it have been under-researched.

Of particular interest to early immune responses, it is now evident that neutrophils and T cells co-exist in the lymph nodes and tissues during health and disease (25–28). Neutrophils migrate to the lymph nodes in response to different stimuli (29) by exiting the circulation through high endothelial venules (HEVs) (30) or *via* the afferent lymphatics (28). Several studies have shown that they can be involved in lasting interactions with other immune cells (28, 31). For example, neutrophils migrate within lymphatic vessels and enter the draining lymph nodes in response to microbial infection during skin inflammation, which subsequently leads to increased lymphocyte proliferation and enhanced adaptive immunity (28). It remains unclear whether or not neutrophils degranulate or undergo any of the processes that lead to the release of their intracellular mediators within the lymph node. However, MPO has been shown to be deposited in the lymph nodes 4 hours after OVA/LPS injection (32) and neutrophil cytoplasts (the remnants of NETs following the expulsion of their DNA) have been identified in the mediastinal lymph nodes of mice in a model of allergic asthma (2). Moreover, we have recently shown that neutrophil-derived cathelicidin can be observed in the lymph nodes of mice immunized with heat-killed *Salmonella typhimurium* (21).

It is critically important to understand the mechanisms behind these neutrophil-T cell interactions and how they may alter T cell function during disease, and in particular to do so using human cells. Deciphering how lymph node-stage or tissue-stage interactions between the two cells lead to altered T cell responses is likewise important. However, deciphering confusing *in vivo* results by performing depletion of neutrophils is difficult as this leads to an increase in T cell-stimulatory cytokine production (33). As a result, *in vitro* co-culture systems are essential for unpicking mechanisms by which neutrophils influence T cell behavior. Co-culture systems also allow us to observe differential effects of resting, de-granulating or NETosing neutrophils using pure populations of cells.

Previous work using such systems has cultured naïve T cells with untouched, freshly isolated peripheral blood neutrophils, usually in the presence of high-dose T cell activation agents. These cultures mimic the interaction of resting neutrophils with early-activating T cells undergoing antigen presentation by dendritic cells in the lymph nodes.

These cultures do not however model other interactions that occur *in vivo*: 1) naïve cell-cell contact in the blood, 2) interaction of T cells with activated neutrophils or their released contents during the circa 24 hours T cells are receiving signals in lymph nodes (28, 34–36) or 3) contact in inflamed tissues or tumors, at which sites the vast majority of T cells present have previously been activated in the lymph nodes and so are not naive.

In addition, neutrophils moving into lymph nodes and tissues during disease are often activated or releasing their contents by de-granulation or NETosis (37). To our knowledge, no *in vitro* paper has modelled the interaction of human T cells with autologous primed neutrophils or their released contents. With this in mind, we aimed to establish a protocol for culturing human neutrophils and T cells. Here, we describe this protocol and assess the impact of differing neutrophils on T cell phenotype and function. We observe that previous demonstrations of neutrophil suppression of T cell responses occur when the T cells are in the early stages of activation. In contrast, if the T cells have previously been activated and meet the neutrophils subsequently, suppression does not occur. We also note that resting, and primed neutrophils, and their contents, differentially affect T cell phenotype. These findings have important implications for understanding the interactions of these cells *in vivo*.

## Methods

### Healthy Human Donors

Peripheral venous blood was collected from healthy adult volunteers under ethical agreement code AMREC 20-HV-069, which included informed written consent. All University of Edinburgh ethical regulations were observed and overseen by the University of Edinburgh Centre for Inflammation Research Blood Resource Management Committee.

Blood was collected into sodium citrate and was processed immediately or within 30 mins of blood draw.

### T Cell and Neutrophil EasySep Isolation

EasySep separation kits (StemCell Technologies, T Cells: #19661, Neutrophils: #19257) were used to isolate CD3^+^ T cells or neutrophils directly from human whole blood by negative selection, as per the manufacturer’s guidelines. Briefly, 50 ul/ml of Isolation Cocktail and 50 ul/ml of magnetic RapidSpheres were added and incubated at room temperature for 5 mins. Samples were then topped up with 1X PBS and placed into an appropriate magnet for 5 mins. The enriched cell suspension was collected and incubated for a further 5 mins with the same volume of RapidSpheres as used previously. Finally, the sample was placed in the magnet for another 5 mins before the final enriched cell suspension was collected.

### Neutrophil Treatments

We assessed the impact of resting, NETotic, and primed neutrophils, as well as neutrophil contents. Total neutrophils were resuspended in 1X PBS at a concentration of 7.5 million/mL before each treatment was carried out.

Resting neutrophils were untreated and cultured with T cells immediately. To direct cells towards NETosis, neutrophils were treated with PMA (Sigma Aldrich, #P1585) for 2.5 hours at 37°C.

Primed neutrophils were obtained by treating the cells for 25 mins with 10 μM cytochalasin B (Merck, #C6762) and 100 nM N-Formylmethionyl-leucyl-phenylalanine (fMLF) (Merck, #F3506); or with 2.5ng/ml LPS (Bio Science #AV-7016-1) or 20ng/ml TNF (RnD Systems #210-TA) for 30 minutes. Cells were then washed well before co-culture. Neutrophil contents were obtained by freeze-thawing the cells 5 times on dry ice, followed by high speed spin to remove cell membranes, as described by Miles et al. (38).

All neutrophils were thoroughly washed with PBS before being co-cultured with T cells.

### Cell Culture

T cells were co-cultured with neutrophils at a 5:1 N:T ratio in round-bottom 96 well plates, in complete medium (RPMI, 10% fetal calf serum, 10 units/mL penicillin, 10 μg/mL streptomycin and 2 mM L-glutamine, all supplied by Gibco, ThermoFisher UK). To model interaction of naïve T cells and resting neutrophils, cells were co-cultured in the absence of any stimulation. To model the interaction of early-activating T cells and neutrophils in the lymph node, cells were co-cultured in the presence of αCD3/αCD28 activator (1 μL ImmunoCult activator to 1x10^5^ cells, StemCell, #10971), which remained in the well throughout culture. To model interaction of late-activating T cells with neutrophils in the inflamed tissue, T cells were first given αCD3/αCD28 stimulation for 24 h in the form of CD3/CD28-coated Dynabeads (1 bead:100 cells; Gibco, #11161D). The beads were then removed with a magnet, the same donor bled again, and fresh autologous neutrophils added as above in fresh medium.

### Proliferation Assay

In order to assess proliferation, T cells were resuspended in 1X PBS and stained with CFSE (Invitrogen, #C34554, working concentration: 5 μM). T cells were incubated at 37°C for 20 mins and then washed twice with an excess of media. Proliferation analysis by dye dilution was performed by flow cytometry following 3 days co-culture.

### Flow Cytometry

Cells were stained for surface markers for 30 mins at 4°C, protected from light. DAPI (Invitrogen, #D1306, working concentration: 1 μg/mL) was added prior to running to assess viability. Samples were analyzed using a LSR Fortessa cytometer (BD Biosciences) and FlowJo software.

### Antibodies

CD4-PE/Cy7 (clone A161A1, Biolegend, #357410, lot B225074); CD8-AF647 (clone HIT8a, Biolegend, #300918, lot B235677); PD1-PE (clone EH12.2A7, Biolegend, #329905, lot B252642).

### ELISAs

The concentration of TNFα (R&D DuoSet ELISA, #DY210), IL-10 (R&D DuoSet ELISA, #DY217B) and IFNγ (R&D DuoSet ELISA, #DY285) in cell culture supernatants was determined by ELISA, as per the manufacturer’s guidelines.

### Statistics

All data shown are expressed as individual data points with line at mean +/- standard error. Statistical analysis was performed using GraphPad Prism software. Two groups were compared with two-way paired Student’s t-tests. Multiple groups from the same experiment were compared using a one-way analysis of variance (ANOVA) test with a Dunnett post-test. A minimum of three donors were used over at least 2 experiments. Details of sample sizes and statistical analyses performed are included in all figure legends.

### Data Sharing Statement

All data are available upon request to the corresponding author at Emily.findlay@ed.ac.uk

## Results

### Establishment of a Neutrophil – T Cell Co-Culture System

**Figure 1:**
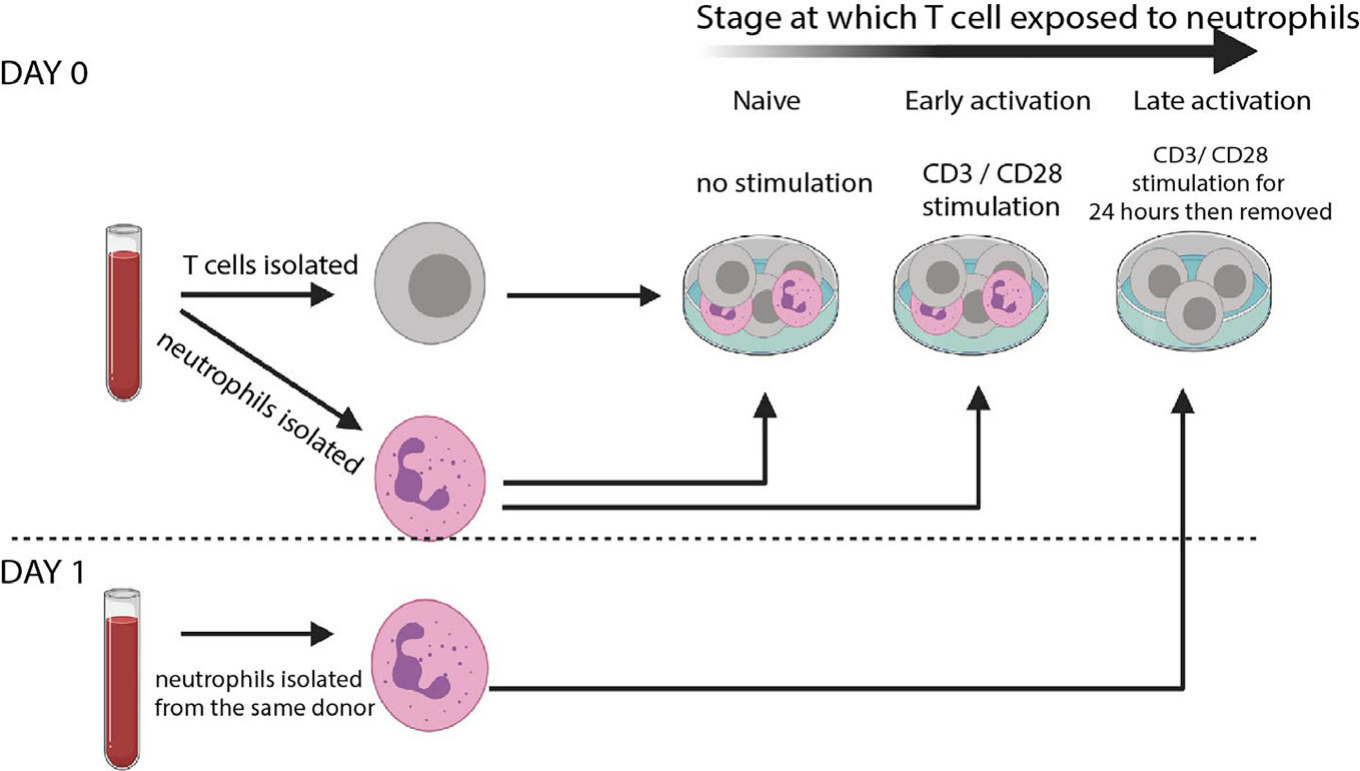
T cell – neutrophil co-culture system. Schematic of culture system. Rapid isolation of peripheral blood neutrophils by negative magnetic selection is performed before autologous T cells are isolated. Cells are co-cultured at a 5: 1 Neutrophil: T cell ratio either without any stimulation (‘naïve’) or in the presence of CD3/CD28 stimulating activation cocktail (‘early’). T cells are also cultured alone in the presence of αCD3 αCD28-coated Dynabeads. 24 hours later Dynabeads are removed and fresh autologous neutrophils isolated. These are co-cultured without any further stimulation (‘late’).

Our protocol is shown in **Figure 1**. T cells and autologous neutrophils are isolated from peripheral blood of healthy donors within 30 minutes of blood draw, by using rapid isolation with magnetic beads. This removes the possibility that extended blood preparation techniques will activate neutrophils or that the cells will begin to die. Another benefit of this technique is that each experiment requires less than 9ml blood.

To model interaction of naïve T cells and resting neutrophils (such as those that occur in the blood), cells are co-cultured without stimulation for 24 hours. To model the interaction of early-activating T cells and neutrophils in the lymph node, in the presence of dendritic cells bearing foreign antigen, cells are co-cultured in the presence of a low dose of αCD3/αCD28 activation cocktail. To model interaction of late-activating T cells with neutrophils in the inflamed tissue, T cells are first given αCD3/αCD28 stimulation for 24 hours in the form of Dynabeads. The beads are then removed with magnets, and fresh autologous neutrophils added. Use of an activation cocktail in the ‘early activating’ condition avoids the use of Dynabeads in the same well as neutrophils, which can inhibit Dynabead action and give the false appearance of suppression (39); however, use of Dynabeads rather than soluble activators for the ‘late activation’ condition allows their complete removal with magnets before neutrophils are added on day 1.

We assessed the impact of resting, primed, apoptotic or NETotic neutrophils, as well as their intracellular contents (38), on T cell phenotype. Activation of T cells could be assessed with flow cytometry, and labelling of T cells with CFSE allowed assessment of proliferation. Finally, supernatants were collected for cytokine analysis.

Our protocol therefore allows analysis of many ways T cells and neutrophils can interact during inflammatory disease. It more closely models *in vivo* situations than any other currently used. Using this protocol, we asked two questions: 1. Do naïve, early-activating and late-activating T cells respond differently to neutrophil contact? And 2. How do T cells respond to resting, NETotic, or primed neutrophils or their contents?

### Early- and Late-Activating T Cells Respond Differently to Neutrophil Contact

**Figure 2:**
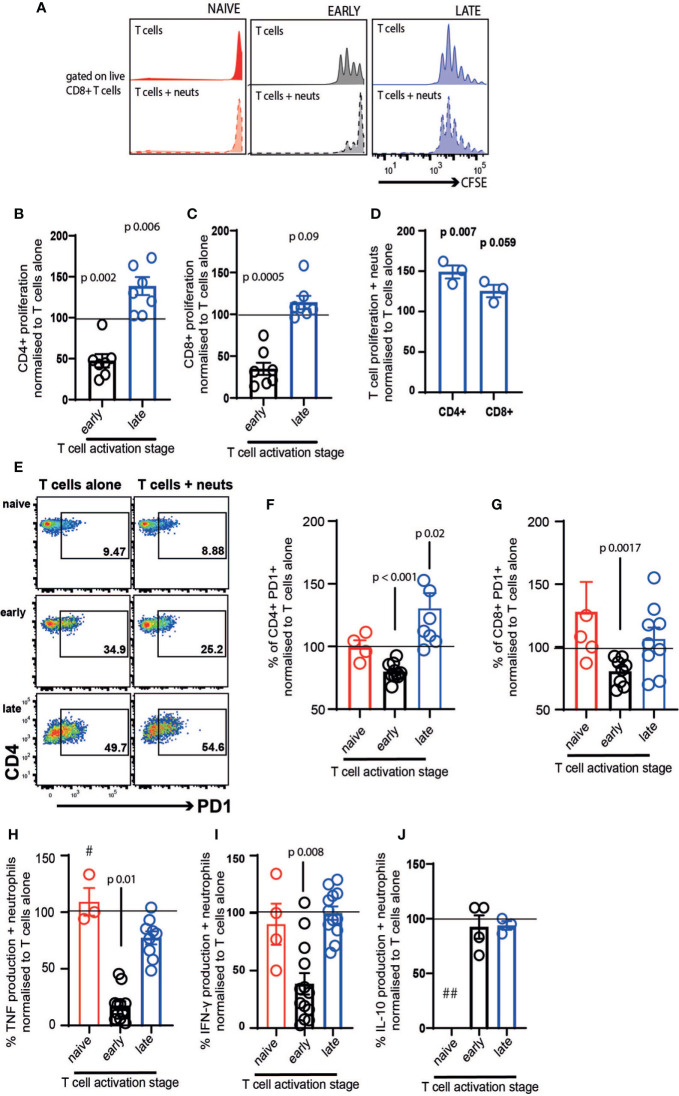
Neutrophil contact differentially affects early- and late-stage activating T cells. T cells and neutrophils were isolated from peripheral blood of healthy human donors using negative magnetic separation, and were cultured together at a 5:1 neutrophil: T cell ratio. **(A)** representative flow cytometry plot and **(B–D)** quantification of proliferation of CD4+ and CD8+ T cells alone and in the presence of resting neutrophils. **(E–G)** Following 24 hours co-culture T cell activation was assessed by flow cytometry analysis of PD-1 and **(H–J)** cell culture supernatant was collected at 24 hours and cytokine production assessed by ELISA. In all cases red symbols = naïve T cells, black = early-activating and blue = late activating. # = two samples were below the limit of detection; ## = all samples were below the limit of detection. N values of individual donors, each of which was plated separately: **(B, C)** 7; **(D)** 3; **(F, G)** naïve 4, early 10 for CD4 and 7 for CD8, late 7 for CD4 and 8 for CD8 **(H)** naïve 3, early 13, late 10; **(I)** naïve 4, early 13, late 12; **(J)** early 4 late 3. Data between T cells exposed to neutrophils and control T cells were analyzed by paired t tests on raw data before conversion to percentages.

We firstly examined the impact of neutrophil exposure on T cell proliferation after 72 hours co-culture. Naïve T cells did not proliferate either in the presence or absence of neutrophils (**Figure 2A**). As previously demonstrated *in vivo* and in other culture models (9, 17, 40, 41), neutrophils suppressed proliferation of early-activating CD4^+^ (**Figure 2B**) and CD8^+^ T cells (**Figure 2C**, black bars). However, this was not the case with late-activating T cells. If the T cells had received stimulation for 24 hours before neutrophil addition, neutrophils had the opposite effect, increasing proliferation of both subsets although most markedly in CD4^+^ cells (**Figures 2B, C**, blue bars). It was possible that this differential response to neutrophils was a consequence not of the neutrophils themselves, but of neutrophil exposure in the presence of continued stimulation. To check this, we performed a second experiment where we removed the Dynabeads from the late T cells, as before, but then added activation cocktail +/- neutrophils. In this experiment we saw that T cells responded to neutrophils in the same way – specifically, late-activating T cells proliferated more following neutrophil exposure, even if antigenic stimulation was ongoing (**Figure 2D**). This also confirmed that, as has been shown previously (39), neutrophils do not suppress the action of soluble CD3/CD28 stimulation on T cells.

Next, we examined the expression of PD1, an early marker of T cell activation (42, 43). Naïve T cells expressed low levels of PD1 after 24 hours in culture, and this was unchanged by neutrophil exposure (**Figures 2E–G**, red symbols). Neutrophils suppressed activation of both CD4^+^ and CD8^+^ T cells in the early-activating cultures, with the proportion expressing PD1 reducing significantly (**Figures 2F, G**). In contrast, neutrophil presence increased the frequency of PD1 expression on late-activating CD4^+^ T cells (**Figure 2F**), and did not suppress expression on CD8^+^ late-activating cells (**Figure 2G**).

Culture supernatants were collected to assess production of inflammatory cytokines following 24 hours co-culture. T cell production of TNF (**Figure 2H**) and IFN-γ (**Figure 2I**) was strikingly reduced by exposure to neutrophils, if it occurred during the early activation process. However, when late-activation stage T cells were incubated with neutrophils, a reduction in cytokine production did not occur. Interestingly, IL-10 production was not altered by neutrophil exposure (**Figure 2J**), suggesting pro- and anti-inflammatory cytokines are affected differentially. In every case, the very low concentration of cytokines produced by naïve T cells was not altered by neutrophil exposure (and IL-10 production by naïve cells was undetectable).

Together, this suggests that early lymph node-stage T cells encountering neutrophils are highly susceptible to suppression of proliferation, activation and pro-inflammatory cytokine production. In contrast, late tissue-stage T cells are not suppressed by neutrophil contact; rather, proliferation and activation of T cells at the ‘tissue-stage’ can be enhanced by contact with resting neutrophils.

### Primed and Resting Neutrophils Induce Opposite Responses in T Cells

Our next question was whether resting or primed neutrophils, or their contents, differentially affected T cells. We firstly examined the activation of T cells exposed to resting neutrophils, those primed with cytochalasin B/fMLF, TNF or LPS, those that were apoptotic or NETotic, or their contents. Neutrophil contents were obtained following lysis by repeated freeze-thaw cycles, which we consider a model of necrotic cell death (38). We used neutrophil contents in our system to compare T cells encountering intact neutrophils to those migrating into an area in which neutrophils have recently died or de-granulated. In the latter situation, we suggest that T cells are exposed to a cocktail of intracellular neutrophil mediators in the absence of any suppressive cell surface receptors.

**Figure 3:**
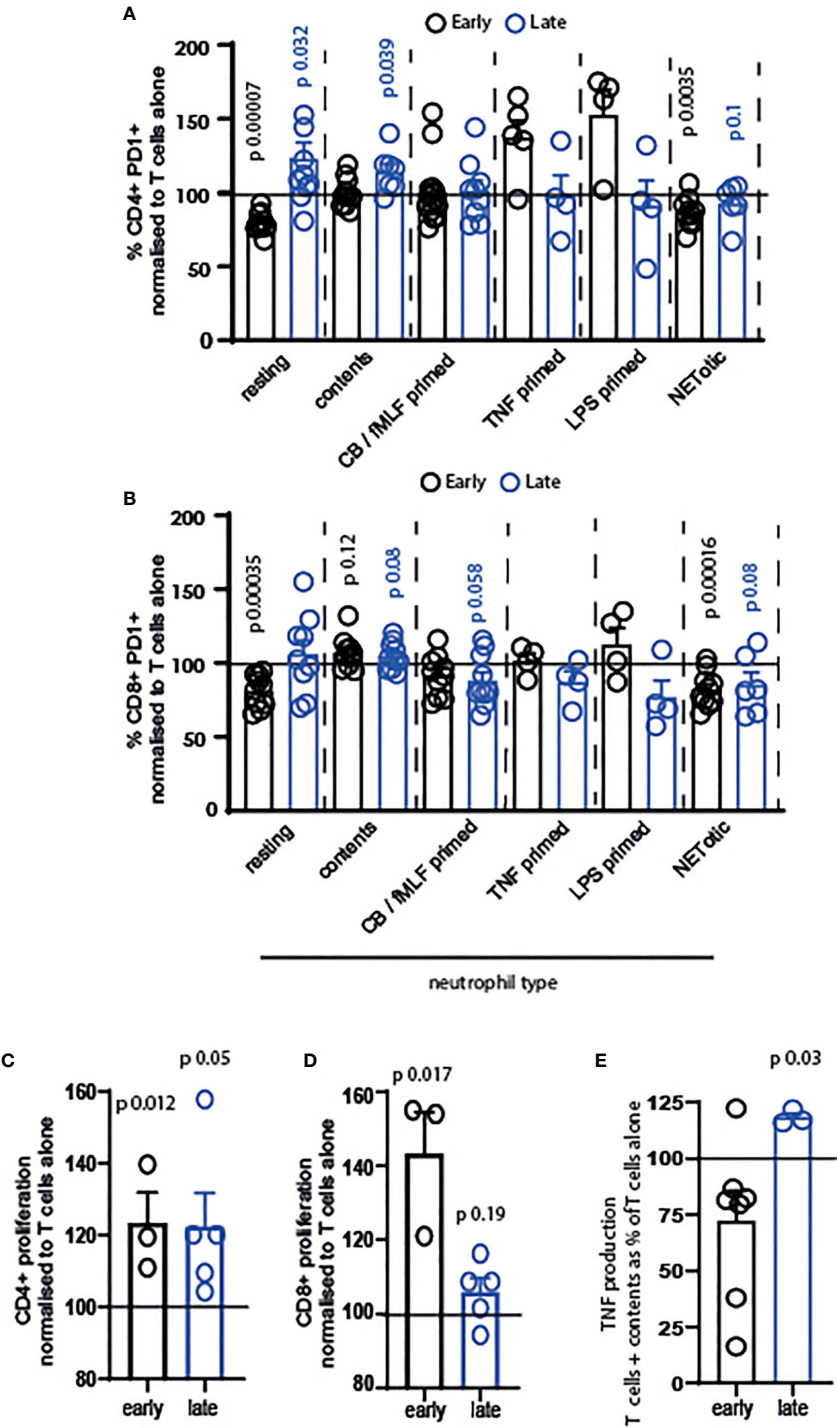
Resting and primed neutrophils, and their contents, differentially affect T cells. T cells and neutrophils were isolated from peripheral blood of healthy human donors using negative magnetic separation, and were cultured together at a 5:1 neutrophil: T cell ratio. Activation of **(A)** CD4+ and **(B)** CD8+ T cells following 24 hours co-culture with neutrophils was assessed *via* quantification of PD1 expression by flow cytometry. Proliferation of **(C)** CD4+ and **(D)** CD8+ T cells was assessed following 72 hours co-culture with contents released from neutrophils during lysing. **(E)** Following 24 hours culture of T cells with released contents, culture supernatant was collected and TNF measured by ELISA. In all cases black symbols = early activating T cells, blue symbols = late activating T cells. N values of individual donors, each of which was plated separately: **(A)** resting 10 early 10 late; contents 10 early 7 late; CB FMLF 14 early 9 late; TNF 5 early 4 late; LPS 4 early 4 late; NETotic 10 early 6 late. **(B)** resting 10 early 9 late; contents 10 early 7 late; CB FMLF 10 early 11 late; TNF 4 early 4 late; LPS 4 early 4 late; NETotic 10 early 6 late. **(C)** 3 early 5 late; **(D)** 3 early 5 late; **(E)** 7 early 3 late. Data between T cells exposed to neutrophils and control T cells were analyzed by paired t tests on raw data before conversion to percentages. CB/FMLF = primed with cytochalasin B and N-Formylmethionyl-leucyl-phenylalanine.

The results of this are intriguing. Firstly, only resting and NETotic neutrophils significantly suppressed activation of early-activating CD4^+^ and CD8^+^ T cells (**Figures 3A, B**, black symbols). In contrast, primed neutrophils did not suppress activation significantly.

Secondly, we observed that neutrophils primed with different mediators have different outcomes on the T cells. While cytochalasin B/fMLF primed cells did not alter early T cell activation, and in fact suppressed late-stage T cell activation (**Figures 3A, B**), neutrophils primed with TNF and LPS increased activation of CD4^+^ T cells in particular (**Figure 3A**). Although this was not a significant difference, owing to variation between donors, the increase in activation of T cells exposed to these primed neutrophils – and the fact that only early cells responded in this way - is interesting.

Finally, we observed that released neutrophil contents increased PD1 expression on late-stage CD4^+^ and both early- and late-stage CD8^+^ T cells. Released contents were therefore the most consistently pro-activatory condition we used.

We were interested in this, and so investigated the impact of neutrophil contents in more detail. Surprisingly, neutrophil contents enhanced the proliferation of CD4^+^ (**Figure 3C**) and CD8^+^ (**Figure 3D**) T cells at both activation stages – that is, in every condition tested. In addition, the late production of TNF (**Figure 3E**) was enhanced.

Together, these data demonstrate that different neutrophils differentially affect T cell activation and proliferation.

## Discussion

We have developed a co-culture system which allows dissection of the interactions between human T cells and autologous neutrophils in a variety of inflammatory settings. Using this system, we have observed a) that T cells which meet neutrophils at the same time as CD3 stimulation react very differently to those that encounter the neutrophils later; and b) that resting, primed, NETotic neutrophils, and their intracellular contents, all differentially affect T cell phenotype and function. These results support previous research showing that neutrophil influence on adaptive immunity is sophisticated and context-dependent; they also demonstrate the importance of carefully planning *in vitro* co-culture systems to investigate observations made *in vivo*.

We conclude firstly that neutrophils have no impact on naïve T cells, as might be expected for cells that meet so frequently in the blood. Next, we show that if resting neutrophils are present early in the T cell activation process, they strongly suppress activation, proliferation and pro-inflammatory cytokine production. However, later on in this process these same cells do not suppress, and can in fact enhance activation of T cells. This has not previously been observed. This study was focused on developing a culture system and validating it with initial observations, rather than mechanistic analysis, and so we do not yet understand why these differences may occur. Current hypotheses center on a) the potential for neutrophils to interact with a cell surface marker on T cells which is only expressed following initial activation; b) potential differences in the activation threshold of T cells (early T cells might have a lower threshold than late ‘tissue-stage’ lymphocytes and neutrophils might provide excessive stimulation, which can lead to inhibition); and c) interaction of neutrophils with cytokines or other mediators released from activated T cells. Deep phenotyping of T cells at the point of neutrophil contact will allow the testing of these hypotheses.

We also established that while resting neutrophils suppress early-activating T cells, if the neutrophils present are primed with TNF, LPS or cytochalasin B/fMLF, they are not suppressive but may in fact promote activation. This suggests a check in the development of an adaptive response, in which neutrophil activatory signals must have been received to promote an inflammatory response but, in their absence, the default is suppression. Proteomic analysis of NETotic, primed, apoptotic, and resting neutrophils may allow determination of how they differentially affect T cell phenotype and function.

Finally, we demonstrate that the release of neutrophils’ intracellular contents enhances proliferation of T cells and, in particular, is strongly pro-stimulatory to CD8^+^ T cells. We used these contents to model the encounter of T cells with mediators released by neutrophils following de-granulation or necrotic cell death in the inflamed tissue. The fact that they induce T cell activation and proliferation has not previously been demonstrated; however, our data supports work showing that individual intracellular neutrophil mediators can promote T cell differentiation and inflammatory cytokine production. For example, lactoferrin promotes Th1 generation in concert with BCG vaccination (44) and increases T cell cytokine production during infection with *Staphylococcus aureus* (45); the neutrophil alpha defensins induce NFkb signalling in T cells (46); and the granule peptide cathelicidin promotes Th17 differentiation (21) and activates CD8^+^ T cells (47). However, all of these interpretations must be made in light of the fact that NETotic neutrophils did not activate the T cells, but rather suppressed as significantly as resting cells. The composition of the two conditions – in particular, the presence of DNA and histone proteins – must be analysed for full interpretation of this phenomenon. Future work will focus on understanding which mediators in particular are responsible for our observed pro-stimulatory effects, through mass spectrometry analysis and mechanistic targeting of those identified.

Our results are intriguing in that they show that CD4^+^ T cells react more consistently to neutrophil co-culture than CD8^+^ T cells. Specifically, early-stage CD4^+^ T cells are significantly suppressed by resting neutrophils while late-stage CD4^+^ cells are activated (**Figure 2F**); both stages are suppressed by NETotic cells; and both stages are induced to proliferate further by released neutrophil contents.

One type of neutrophil which we did not investigate was apoptotic cells. Apoptotic neutrophils can directly and indirectly modulate the adaptive immune response. For instance, DCs can take up antigens from apoptotic neutrophils and act as professional APCs for T cells (48). The release of epidermal growth factor by apoptotic neutrophils is also involved in the maturation of monocytes into DCs, subsequently promoting the activation of anti-viral CD8^+^ T cells (49). Furthermore, engulfment of apoptotic neutrophils regulates IL-23 cytokine production by phagocytes, thereby controlling T cell-derived IL-17 production (50). Conversely, uptake of human apoptotic neutrophils by DCs has also been shown to impair DC co-stimulation and antigen presentation, resulting in diminished allogeneic T cell responses (51). Apoptotic PMN that have taken up tumor cell-released autophagosomes display enhanced immunosuppressive functions: they inhibit the proliferation and activation of both CD4^+^ and CD8^+^ T cells in a process requiring cell-contact and the generation of ROS (52). In addition, human neutrophils can induce development of regulatory T cells *via* apoptotic bodies (53). Extending our observations to apoptotic neutrophils and comparing those results to NETotic cells may help to determine the mechanistic pathways involved.

Here we have demonstrated that neutrophils can suppress or activate T cells in an opposite manner depending on whether the T cells are naïve, receiving CD3 stimulation or at a later activation stage. This both supports previous published work (which shows neutrophil suppression) but also extends it considerably. Our observations reveal a more sophisticated T cell – neutrophil interaction than previously observed and open a new focus for research, as lymph node and tissue (or tumour) T cells may respond completely differently to the same neutrophil encounter.

To our knowledge, this is also the first demonstration that neutrophil released contents have opposite outcomes on T cell phenotype compared to resting neutrophils. This suggests that T cells encountering an area of neutrophil necrosis, and consequent mediator release, will respond differently to those encountering intact neutrophils with cell surface proteins present.

Together, our work confirms the need for nuanced culture models which recapitulate each stage of inflammatory disease with accuracy, and may explain why *in vitro* systems used previously do not always explain observations made *in vivo.*

## Data Availability Statement

The raw data supporting the conclusions of this article will be made available by the authors, without undue reservation.

## Ethics Statement

The studies involving human participants were reviewed and approved by University of Edinburgh internal review. The patients/participants provided their written informed consent to participate in this study.

## Author Contributions

DM, KS, and EG performed experiments. AR provided resources. DM, GH, AR and EG analyzed data and wrote the manuscript. All authors contributed to the article and approved the submitted version.

## Funding

This work was funded by a Royal Society Dorothy Hodgkin Fellowship (DH150175) and a Tenovus Scotland project grant (E17/01) to EG and a Medical Research Council project grant (MR/K013386/1) to AR.

## Conflict of Interest

The authors declare that the research was conducted in the absence of any commercial or financial relationships that could be construed as a potential conflict of interest.
